# Ecosystem Services and Biodiversity in a Rapidly Transforming Landscape in Northern Borneo

**DOI:** 10.1371/journal.pone.0140423

**Published:** 2015-10-14

**Authors:** Nicolas Labrière, Yves Laumonier, Bruno Locatelli, Ghislain Vieilledent, Marion Comptour

**Affiliations:** 1 UPR Biens et services des écosystèmes forestiers tropicaux (BSEF), Centre de coopération internationale en recherche agronomique pour le développement (CIRAD), Montpellier, France; 2 Ecole doctorale ABIES, AgroParisTech, Paris, France; 3 CGIAR Research Program – Forests, Trees and Agroforestry, Center for International Forestry Research (CIFOR), Bogor, Indonesia; 4 CGIAR Research Program – Forests, Trees and Agroforestry, Center for International Forestry Research (CIFOR), Lima, Peru; 5 Centre d’Ecologie Fonctionnelle et Evolutive (CEFE), CNRS, Université Montpellier II, Montpellier, France; National University of Singapore, SINGAPORE

## Abstract

Because industrial agriculture keeps expanding in Southeast Asia at the expense of natural forests and traditional swidden systems, comparing biodiversity and ecosystem services in the traditional forest–swidden agriculture system vs. monocultures is needed to guide decision making on land-use planning. Focusing on tree diversity, soil erosion control, and climate change mitigation through carbon storage, we surveyed vegetation and monitored soil loss in various land-use areas in a northern Bornean agricultural landscape shaped by swidden agriculture, rubber tapping, and logging, where various levels and types of disturbance have created a fine mosaic of vegetation from food crop fields to natural forest. Tree species diversity and ecosystem service production were highest in natural forests. Logged-over forests produced services similar to those of natural forests. Land uses related to the swidden agriculture system largely outperformed oil palm or rubber monocultures in terms of tree species diversity and service production. Natural and logged-over forests should be maintained or managed as integral parts of the swidden system, and landscape multifunctionality should be sustained. Because natural forests host a unique diversity of trees and produce high levels of ecosystem services, targeting carbon stock protection, e.g. through financial mechanisms such as Reducing Emissions from Deforestation and Forest Degradation (REDD+), will synergistically provide benefits for biodiversity and a wide range of other services. However, the way such mechanisms could benefit communities must be carefully evaluated to counter the high opportunity cost of conversion to monocultures that might generate greater income, but would be detrimental to the production of multiple ecosystem services.

## Introduction

Drastic land-use transformations have occurred in the tropical forest landscapes of Southeast Asia in the past decades, leading to the disappearance of natural forests and the replacement of traditional land-use systems with monoculture plantations. On the island of Borneo, the lowland rainforests are at the crossroads of multiple and divergent interests. While these rainforests are hotspots of biological diversity with a high rate of endemism and hold important carbon stocks, they are also a major source of valuable timber, and are situated on lands that are very suitable for conversion to oil palm or other large industrial plantations [1–3].

Since the late 1960s, logging has affected most of the lowland forests [4]. Following the boom era that lasted roughly until the 2000s, large areas of logged-over forest were left unmanaged. Although several studies demonstrated the important role that these forests play in supporting biodiversity and maintaining multiple ecosystem services [5–8], they were slowly depleted through illegal logging and finally converted to oil palm plantations [9]. The detrimental effect of such large-scale land clearing on biodiversity and other services is an accepted premise [10–13]. At the same time, as the extent of industrial agricultural areas keeps increasing, the role of traditional agricultural systems (swidden i.e., slash-and-burn and rotational fallow farming, and smallholder agroforestry systems) vs. alternative agricultural systems in providing goods and services has received much attention [14–17]. To date, however, there has been little consensus about their role in supporting biodiversity and producing ecosystem services.

Since negative correlations usually exist between goods and services (e.g. [18,19]), human-modified land-use areas would not be expected to produce levels of services similar to those of natural forests. Yet, in Sumatra, under low management intensity conditions, mature rubber gardens were found to have a plant species richness similar to that of nearby natural or secondary forests and to store substantial amounts of carbon in aboveground biomass [15,20]. Swidden fallows were also shown to reduce soil erosion and contribute to soil nutrient cycling to levels similar to those found in natural forests [21–23]. In contrast, studies in West Kalimantan found that an increasing number of shifting cultivation cycles naturally led to a decrease in tree species richness and important tree composition changes [24]. Some argue that such human-modified land use will not allow any long-term conservation goal to be fulfilled, partly because maintaining tree diversity might hinder rubber garden productivity [25]. Overall, despite current debates about the capacity of human-modified landscapes to protect biodiversity and support ecosystem services, these landscapes are getting increasing attention for their contribution to biodiversity conservation in the global context of vanishing natural habitats [26,27].

Research gaps concerning the effect of land-use changes on the ecosystem services produced by swidden systems have been identified through a systematic review currently under progress that aims to bring unbiased evidence to the debate [28]. While demand for food and goods is growing worldwide, and biodiversity and services are being lost [29], such information is essential to building sound land management strategies and guiding decision making on land-use planning.

In this study, we address the following question: What level of biodiversity and ecosystem services are found in the different land uses related to the traditional forest–swidden agriculture system? We conducted a case study from a northern Bornean agricultural landscape where we quantitatively estimated the contribution of various land uses to: (1) climate change mitigation through carbon storage in live aboveground biomass and topsoil, (2) tree species diversity, and (3) soil erosion control. The two services were chosen because of their relevance for multiple beneficiaries at different scales (local to regional for soil erosion control, and global for climate change mitigation). Tree species diversity, which we did not consider as an ecosystem service, was chosen because of its cross-cutting and cross-scale nature, as it jointly influences the delivery of goods (e.g. food, raw material, fruit, and timber for local people) and services (e.g. water regulation at the regional scale) [30].

## Materials and Methods

### Ethics statement

This study strictly complied with Indonesian laws. Authorizations to carry out research activities were obtained from appropriate sources both at national (Ministry of Forestry and Ministry of State for Research and Technology) and local level (Head of Kapuas Hulu regency). Permissions from local owners to work on their lands were obtained prior to any activity. Sending soil and herbarium samples from the field site to laboratories in Java for further analysis was done after authority approval. We did not collect endangered plants or any animals.

### Study site and plot selection

**Fig 1:**
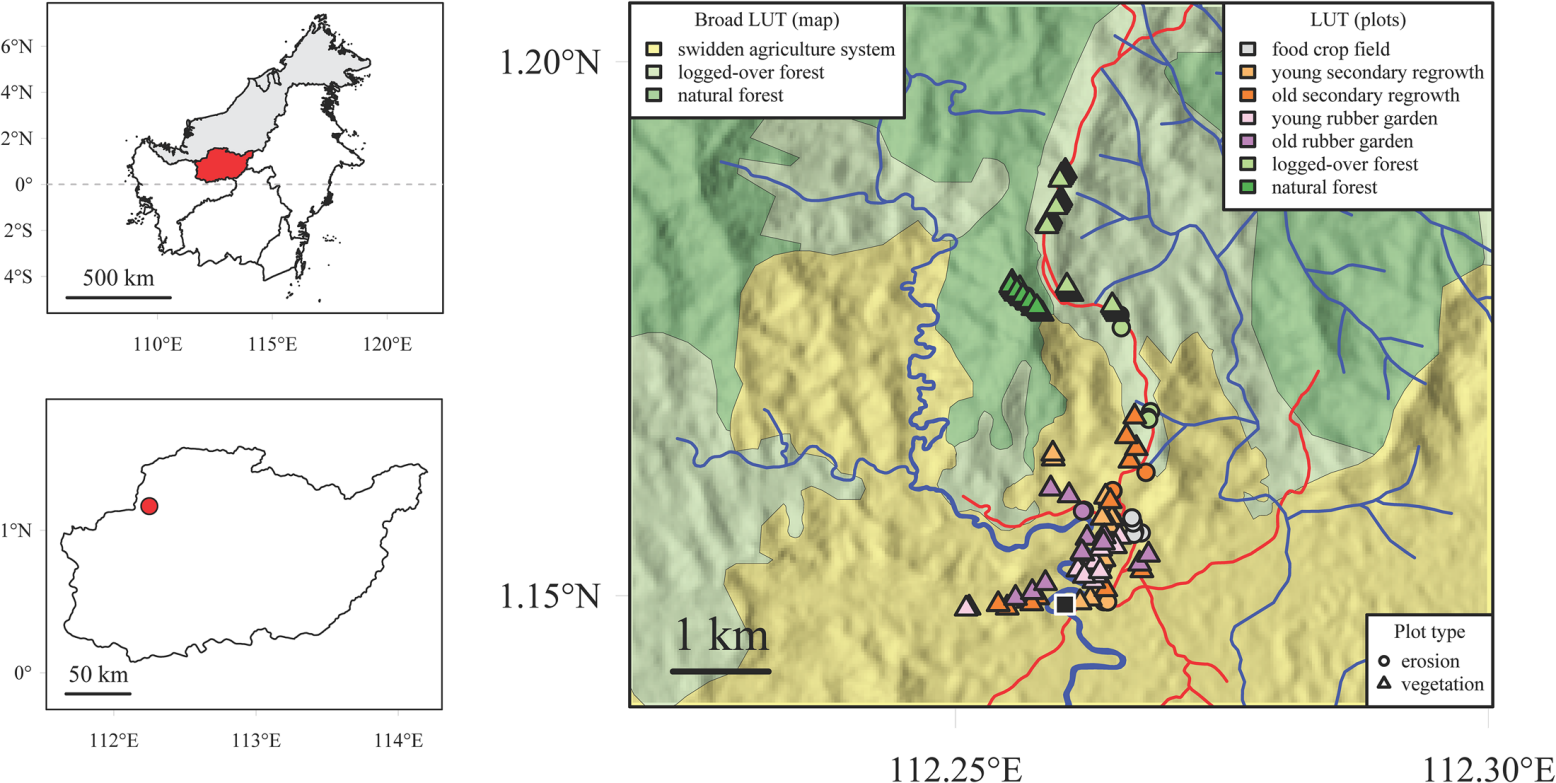
Plot location within the study area. The study area is located on the island of Borneo (top left panel), in the Indonesian province of West Kalimantan, in the regency of Kapuas Hulu (bottom left panel). In the main panel, plot location and broad land-use types (LUT) are displayed along with former logging roads (red) and rivers (blue). The black square indicates the location of the village.

Field work was carried out in the surroundings of Keluin (1°08'57" N, 112°15'37" E), a village located in the district of Batang Lupar, Kapuas Hulu regency, West Kalimantan province, Indonesia (Fig 1). This village is located near a river flowing directly toward the Danau Sentarum National Park, a very complex hydrological system that regulates the hydrological regime of the Kapuas River (the longest river in Borneo, which supplies water to the West Kalimantan capital city of Pontianak) [31]. Altitude in the study area ranges from 50 to 450 m above sea level [32]. Soils have developed over sedimentary rocks [33] and belong mostly to the Ultisols order [34]. Mean annual rainfall is 3300 mm (WorldClim data, interpolated estimate for the 1950−2000 period with a 30 arc-second resolution [35]). The study area has a tropical rainforest climate with a drier period from June to August, but monthly rainfall is highly variable throughout the year.

This site was chosen because of the diversity of land uses representative of northern Bornean traditional agricultural systems that were found within a limited perimeter (ca. 2 km W–E by 5 km N–S). Traditionally, the main crop, rice, is cultivated along with other annual crops (such as cassava, maize, etc.) in swiddens from either primary or secondary forest clearing before the plot is abandoned after 1−2 years of cultivation. Rubber seedlings and saplings, which are planted in some crop fields during the cultivation phase or just after plot abandonment, eventually lead to rubber-based secondary forests (also called “jungle rubber” gardens) as a result of plant succession. Past and current uses of land by the local community (swidden agriculture system mixed with rubber gardens) and past logging activities have created a mosaic of vegetation reflecting various disturbance types, ages and intensities in the Keluin area.

We defined seven land-use types in the study area: (1) food crop fields where the main crop, rice (*Oryza sativa* L.), is planted after using the slash-and-burn method on the initial vegetation (natural forest or secondary vegetation), (2) young secondary regrowth following field abandonment after crops have been cultivated for 1 or 2 years, (3) old secondary regrowth forests further into the process of vegetation succession following field abandonment, (4) young rubber gardens resulting from the planting of rubber seedlings and saplings (*Hevea brasiliensis* (Willd. ex A.Juss.) Müll.Arg.) in some young fallows, (5) old rubber gardens with complex stand structure, (6) logged-over forest that was selectively logged from 1997 to 2005, and (7) natural forest with very little human disturbance.

Two distinct sets of vegetation and erosion plots were established based on a multi-stratified sampling first across land-use type and second across disturbance age for stands regenerating after slash and burn (see S1 Table). For the purpose of this study, young and old stands (≤ 20 years and > 20 years following disturbance, respectively) were distinguished from each other because stand structure becomes more complex 20 years after initial disturbance [36,37]. Plots for the vegetation sampling were scattered across the study area to encompass the variability of topographical situation (ridge, slope, valley bottom) and slope steepness (from flat to very steep) present in this low-elevation hilly landscape. Plots for the erosion protocol were also scattered across the study area, with the additional constraint of having a homogenous slope of ca. 40% (ca. 22°). We chose relatively steep slopes (compared with the standard slope, i.e. 9%, of a typical erosion monitoring method such as the Wischmeier plot [38]), as erosion hardly occurs on non-bare slopes of low steepness in the study region [22].

### Vegetation plots: estimating aboveground carbon stocks and tree species diversity

For young and old stands in both secondary regrowth areas and rubber gardens, 12 plots each of 20 × 20 m were randomly selected (for a total surveyed area of 0.48 ha for each of the four land-use types) to capture the variability of vegetation structure and composition within each land-use type (Fig 1 and S1 Table). For natural and logged-over forests, we surveyed a total area ca. twice as large as that of secondary regrowth areas and rubber gardens to encompass an even much higher variability of vegetation structure and composition: five rectangular plots each of 20 × 100 m (longer dimension along the contour line; 1 ha each forest type) were selected. Plots were contiguous but staggered down the slope for natural forest (all fitting in a 100 × 400 m area). For logged-over forest, they were scattered on either side of a former logging road built on a ridge, ca. 20 m downslope from the road.

All trees with diameter at breast height (DBH, 1.3 m) ≥ 5 cm were measured, tagged and mapped, and their height estimated, following standard procedures [39]. Leaf samples were collected at least once for each vernacular name (consistently given by the same group of highly knowledgeable local people using the Iban language) for both young and old secondary regrowth areas and rubber gardens, and for every individual tree for natural and logged-over forests. Identification of the herbarium vouchers were carried out at the Herbarium Bogoriense in Bogor, Indonesia.

We used three different indices to characterize tree species diversity at the plot level: species richness, Berger–Parker index, and Fisher's α. Species richness is the simplest measure of species diversity but does not take into account community evenness. Conversely, the Berger–Parker index (defined as the inverse of the proportion of individuals of the most common species in the community) depends only on evenness, and is sensitive to the dominance of a few species. Fisher's α is mathematically unrelated to the first two indices, is relatively independent of sample size, and is insensitive to the presence of rare species [40].

We used the generic Chave et al. allometric equation for tropical forests [41] to calculate tree dry biomass. Wood specific gravity, a multiplier included in the aforementioned equation, was obtained from the Global Wood Density Database [42,43]. When species were not found in the database, the genus-level average wood density was used instead. Aboveground biomass (AGB) was split into four fractions (according to tree DBH; 5–10 cm, 10–30 cm, 30–50 cm, and > 50 cm) of which relative proportions were computed with no other analytical purpose than to identify the lowest and highest contributing fractions to total aboveground carbon stocks. Those were calculated from biomass values by application of the standard 0.47 conversion factor [44].

Because of differences in sampling design (inter-plot distance ranged from 20 to 4650 m), we could not use tree diversity values aggregated over the total surveyed area (0.48 ha each for secondary regrowth areas and rubber gardens, 1 ha each for natural and logged-over forest) to compare the different land-use types (see S1 Fig). Instead, we computed individual values–for tree diversity and aboveground carbon–for each 20 × 20 m plot (12 plots each for young and old secondary regrowth areas and rubber gardens, and 25 each for natural and logged-over forests; 98 plots in total) and used resulting mean values to characterize each land-use type. All details about individual plot coordinates, stand age (whenever relevant) and indicator–aboveground carbon and tree diversity–values can be found in S2 Table.

### Erosion plots: soil loss monitoring and topsoil carbon stock estimation

Silt fences (made from a nonwoven polyester geotextile) were used to measure hillslope erosion. Following guidelines from Robichaud and Brown [45], 4-meter-wide fences were set up across the slope, and heavy logs were positioned 15 m upslope from the fences to form the upper boundaries of laterally unbounded plots of ca. 60 m² contributing areas. Fences from 35 plots in total (five replicates for each of the seven land-use types) were cleaned monthly during 15 continuous months (from June 2012 to September 2013), and the collected material was dried, sieved (with a 1 mm sieve) and the weight of the resulting fine mineral fraction was recorded. Composite soil samples from four sampling points per plot (close to each plot corner) were taken for topsoils (0−20 cm). Dried samples (drying temperature T = 105°C) were analyzed for carbon content (Walkley and Black method, [46]). In addition, for each plot, one sample of topsoil (using a 100 cm^3^ ring) was taken at mid-slope and dry bulk density (in g cm^–3^) was measured. Topsoil carbon stocks were then calculated using carbon content and dry bulk density. All details about individual plot coordinates, stand age (whenever relevant) and indicator–topsoil carbon and annual soil loss–values can be found in S3 Table.

### Statistical analysis

All statistical analyses were done using R 3.1.2 [47]. Analyses were done on original values in case of normal data distribution and log_10_-transformed values if transformation led to normal distributions. For each of the six indicators we studied (aboveground carbon, topsoil carbon, annual soil loss, tree species richness, Fisher’s α and Berger-Parker index), we tested for spatial autocorrelation on both initial values and residuals of a linear model against land-use type using Moran’s I. In case residuals were still spatially correlated, we used the Lagrange Multiplier diagnostics for spatial dependence to determine the structure of the appropriate spatial regression model (i.e., spatial error model that accounts for error term correlation vs. spatial lag model that accounts for non-independence between observations; [48]) using various functions from the spdep package [49,50]. We tested for differences (at p < 0.01) in indicator values depending on land-use type using analysis of variance (ANOVA) followed by Tukey’s honest significant difference (HSD) test on either (1) uncorrected values in case indicators or linear model residuals were non-spatially autocorrelated (which was the case for topsoil carbon, annual soil loss and Berger-Parker index), or (2) values corrected from spatial auto-correlation (by subtracting the “signal” term originating from spatial regression to the uncorrected value; [48]). Values of all statistical tests can be found in S4 Table.

A nonmetric multidimensional scaling (NMDS) analysis was performed to illustrate plot similarity in terms of tree species composition (using the metaMDS function; see [51]). NMDS analysis is an ordination technique aiming at iteratively collapsing multidimensional information (in this case, plot species composition) into an optimal—lower—number of dimensions while conserving the rank order of distances [52]. The closer the points in the initial and resulting spaces, the more similar are the tree species compositions of the corresponding plots. Using Spearman's rank-order correlation, we tested for correlations between plot distance in the field and in the NMDS plot to assess to what extent spatial autocorrelation influenced results from the NMDS analysis.

## Results

### Tree species diversity and composition

**Fig 2:**
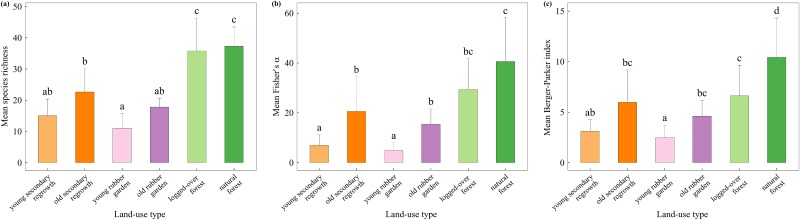
Tree species diversity indices depending on land-use type: (a) species richness; (b) Fischer’s α; (c) Berger–Parker index. Indices were computed for each 20 × 20 m plot before being averaged by land-use type (over 25 plots for logged-over and natural forest, and 12 otherwise). Mean values with the same letter are not significantly different (Tukey’s HSD test on uncorrected values in case no spatial autocorrelation was detected and corrected values otherwise, p < 0.01).

The three species diversity indices were highest in natural forests (Fig 2). However, differences in mean species richness and mean Fisher’s α between natural and logged-over forests were not significant. Only Berger–Parker index values (exclusively dependent on community evenness) were significantly different between these two land-use types. Similarly, natural and logged-over forests had ca. 60% more species than do old secondary regrowth forests (next most species-rich land-use type).

Older stands (for rubber gardens and secondary regrowth forest) consistently showed higher index values compared with young ones, but the difference was only significant for mean Fisher’s α. For similar time intervals since last disturbance, all the diversity indices were consistently higher in secondary regrowth areas compared with rubber gardens, but the difference was not significant.

**Fig 3:**
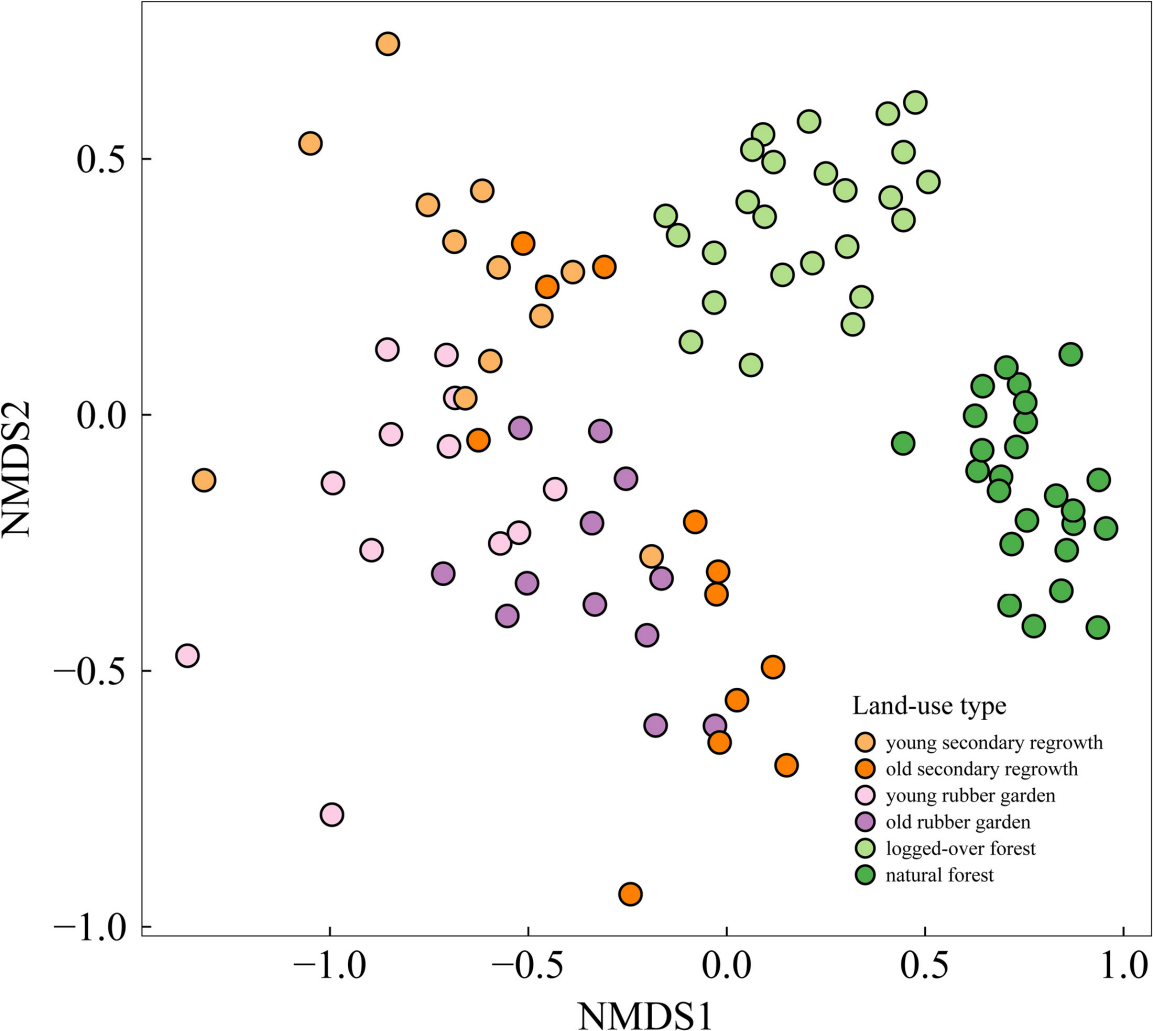
Nonmetric multidimensional scaling (NMDS) plot showing tree species composition similarity among plots under different land-use regimes. Each dot (98 in total) represents a 20 × 20 m plot.

In the two dimensions of the NMDS plot (Fig 3), tree species composition was markedly different in natural forest plots compared with other plots. Logged-over forests had the most similar tree species composition to natural forests. Tree species composition was highly variable among secondary regrowth areas and rubber gardens (both young and old). We found a moderate positive correlation between plot distance in the field and in the NMDS plot (Pearson's *r* = 0.58; p < 0.001). Even though point clustering for logged-over and natural forest might therefore result in part from the sampling design (spatial autocorrelation), a careful inspection of the species present in the different land-use types (see S5 Table) strongly supports our finding that natural forest species are highly specific and different to all other land-use types.

### Carbon storage

**Fig 4:**
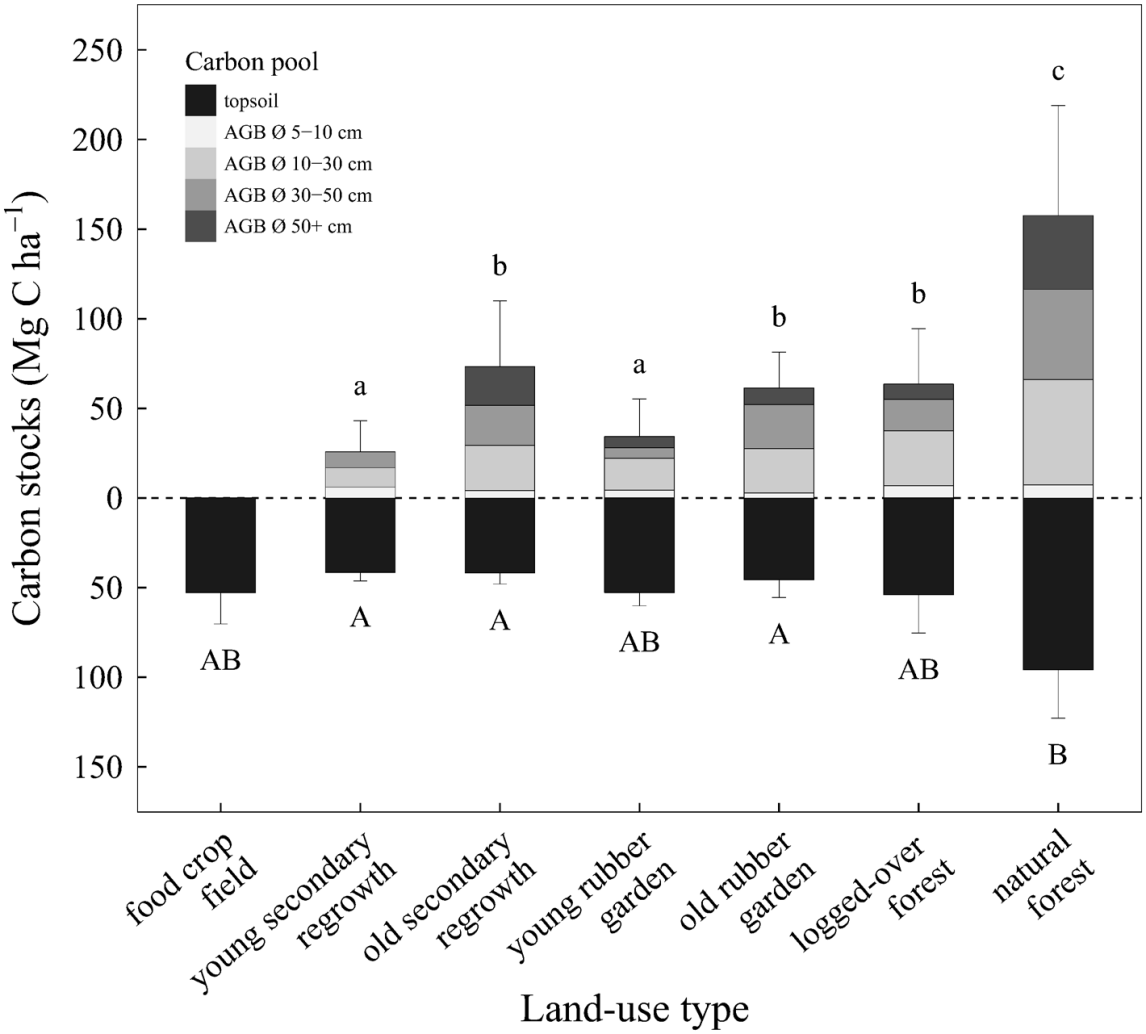
Mean carbon stocks (+ 1 SD) in topsoil (0–20 cm) and aboveground biomass. Means were computed over 12 to 25 replicates per land-use type for aboveground biomass, and over five replicates per land-use type for topsoil. Aboveground biomass (AGB) was split into four fractions according to tree diameter at breast height (Ø). Mean values with the same letter (lowercase for aboveground biomass, uppercase for topsoil) are not significantly different (Tukey’s HSD test on uncorrected values in case no spatial autocorrelation was detected and corrected values otherwise, p < 0.01).

Aboveground carbon stock levels were significantly higher in natural forests than in any other land-use types (Fig 4). Unsurprisingly, the lowest values were found in young stands (rubber gardens or secondary regrowth areas). Even old rubber gardens, old secondary regrowth forests, and logged-over forests had aboveground carbon stocks at levels half of those of natural forests.

For all types of land use, the highest proportions of aboveground carbon were contained in trees with DBH = 10−30 cm, while the lowest non-null proportions were contained in trees with DBH = 5−10 cm. Yet, for recently disturbed stands such as those in young secondary regrowth areas, small trees (those with DBH = 5−10 cm) could represent up to ca. 25% of aboveground carbon.

Although natural forests stored on average twice as much carbon in topsoils as other land-use types, significant differences were observed only between natural forests and secondary regrowth areas (both young and old) or old rubber gardens due to high variability within land-use types (for logged-over and natural forests, especially).

### Relationship between aboveground carbon stocks and species richness

**Fig 5:**
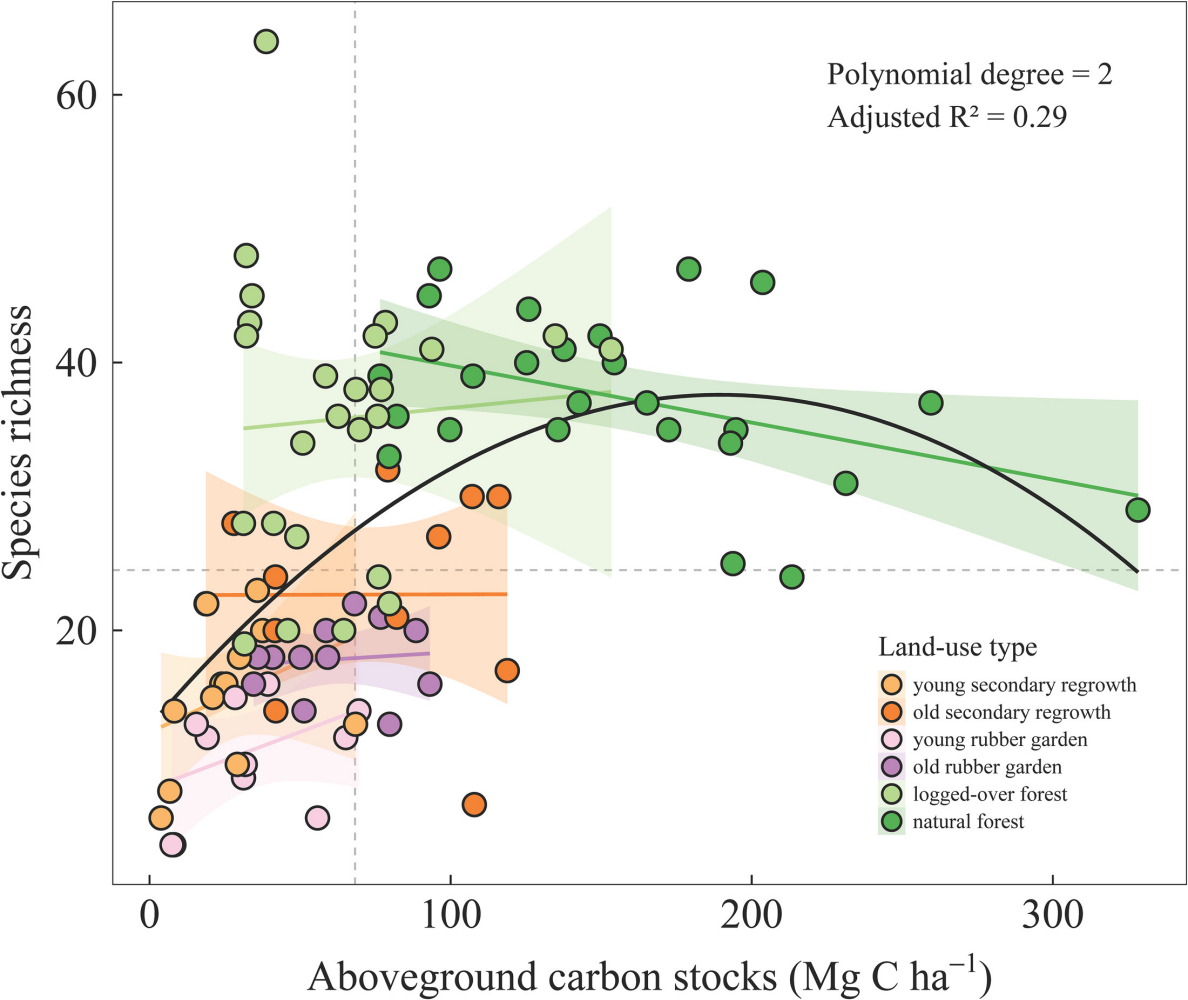
Species richness against aboveground carbon stocks. Each dot (98 in total) represents a 20 × 20 m plot. Horizontal and vertical dashed lines represent median values of species richness (n = 25) and carbon stocks in aboveground biomass (68 Mg C ha-1), respectively. Regression lines (along with standard error) are computed independently for each land-use type. A second-order regression model (best-fit significant model selected among polynomial models with degrees 0 to 3) over the whole data set is also displayed (in black).

The greater the aboveground carbon stocks, the greater the species richness in all land-use types, with the noticeable exception of natural forests for which the relationship is negative (cf. regression lines; Fig 5). Almost all plots with high tree diversity (richness higher than median) and low carbon (aboveground carbon stock below median) belonged to logged-over forests. The vast majority of young secondary regrowth areas and rubber garden plots had low diversity and low carbon. In contrast, all (but one corresponding to a tree-fall gap) natural forest plots showed high diversity and high carbon.

### Soil erosion

**Fig 6:**
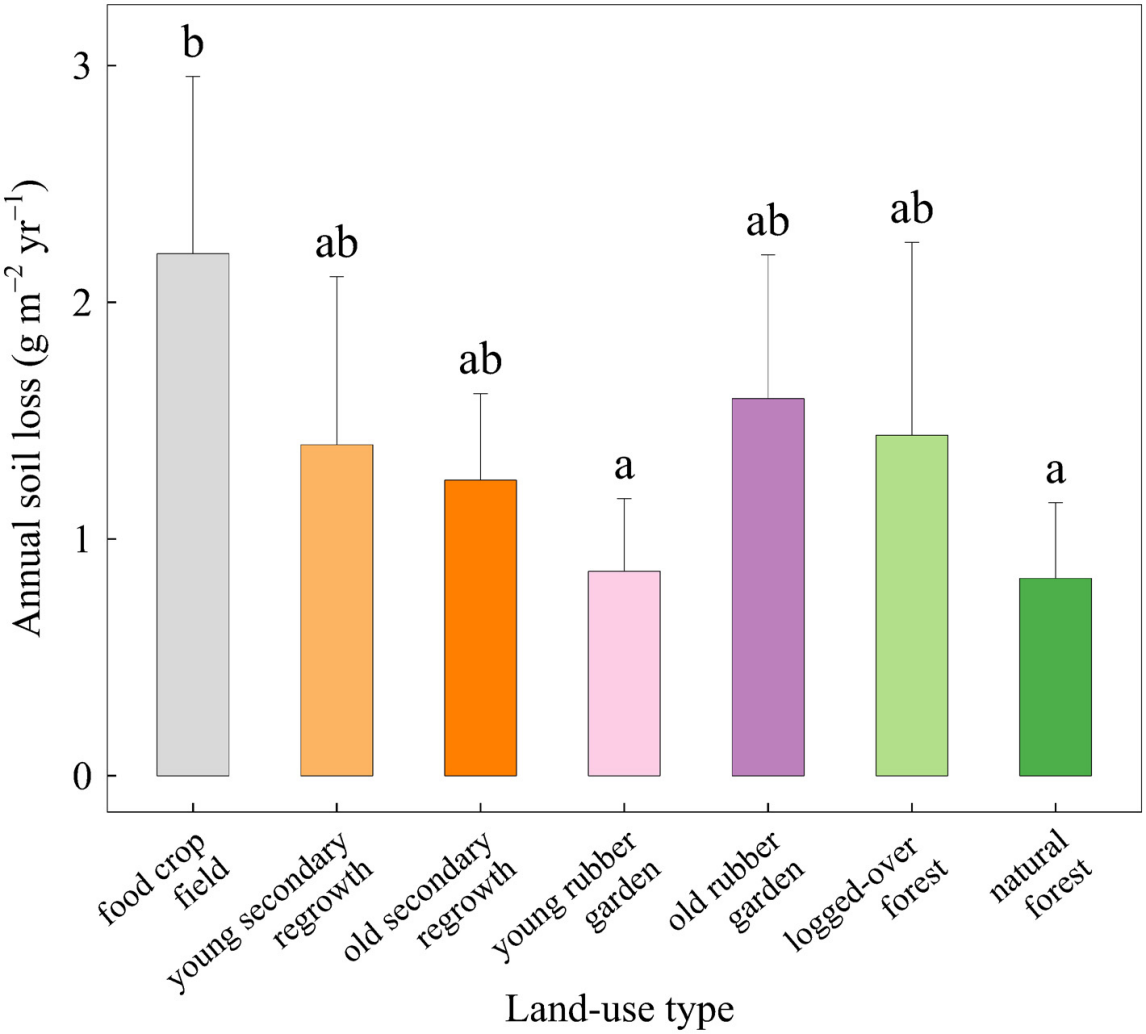
Mean annual soil loss (+ 1 SD) depending on land-use type. Data are averaged over the monitoring period (June 2012 to September 2013) and over the different replicates for each land-use type. Values from three replicates (one in young secondary regrowth area, one in young rubber garden, one in logged-over forest) were discarded because they were abnormally high (> two times mean value of the corresponding land-use type). Mean values with the same letter are not significantly different (Tukey’s HSD test on uncorrected values in case no spatial autocorrelation was detected and corrected values otherwise, p < 0.01).

Annual soil loss values ranged from 0.8 to 2.2 g m^–2^ yr^–1^, with individual plot values varying from 0.5 to 2.7 g m^-2^ yr^-1^ (see S3 Table). Annual soil loss was significantly lower in natural forests and young rubber gardens compared with food crop fields (Fig 6). Other differences in annual soil loss between land-use types were not significant.

## Discussion

### Service production is highest in natural forest

**Fig 7:**
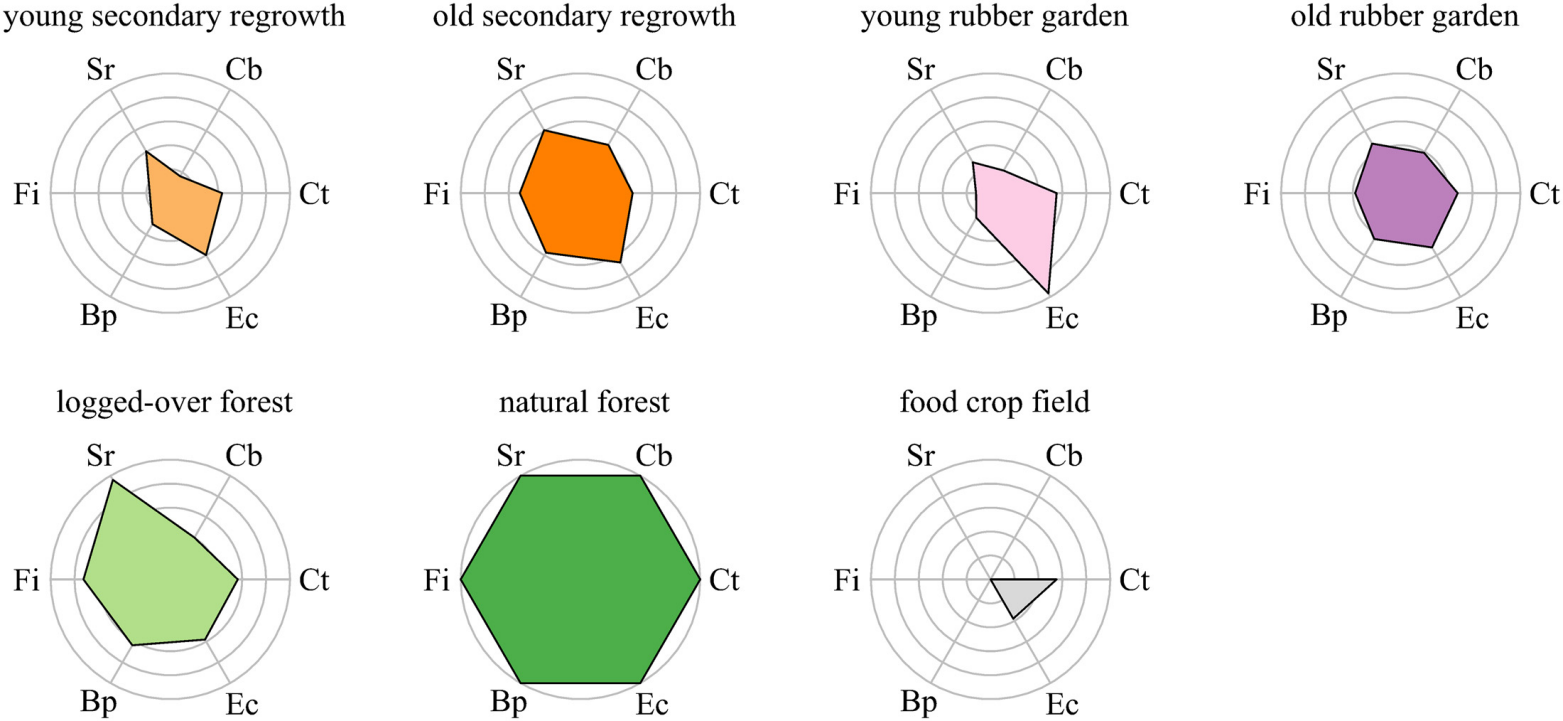
Spider chart of normalized service indicators for different land-use types. Indicators are normalized so that the minimum possible value of an indicator is at the center of the radial plot and the maximum observed values are on the outer circles (for the service of soil erosion control, the indicator is the inverse of the measured soil loss).Service indicators: Ct = carbon stocks in topsoil; Cb = carbon stocks in aboveground biomass; Sr = tree species richness; Fi = Fisher’s α; Bp = Berger–Parker index; Ec = soil erosion control.

As expected, service production was highest in natural forests (Fig 7). Natural forest plots clearly had high levels of both aboveground carbon stocks and tree species richness (top right corner, Fig 5), even though lower tree species richness was observed for the highest values of aboveground carbon stocks. This negative trend might be explained in light of Connell’s intermediate disturbance hypothesis [53]. According to this hypothesis, an intermediate level of disturbance is required for a given tree community to reach maximum species richness [53]. Natural forest succession will lead to the competitive exclusion of early- and mid-successional species, therefore reducing overall species diversity. Our results corroborate this hypothesis because aboveground carbon stocks were positively correlated to disturbance age [54].

Aboveground carbon stocks in human-modified land-use types did not reach half those of natural forests. Logged-over forests, agroforests and secondary regrowth areas had topsoil carbon stocks 40–60% lower than those of natural forests. This contrasts with results from Kessler *et al*. who found no significant reduction in soil carbon stocks between natural forests and cocoa agroforests in Sulawesi, Indonesia [55]. This might in part be due to the fact that cocoa agroforests in their study region are obtained through gradual thinning of the natural forest with minimum impact on the root system [55], while transitions related to practices in our study area (logging, slash and burn) are more abrupt and therefore potentially more disturbing for topsoil.

Regarding soil erosion control, even if soil loss was lowest in natural forests and differed significantly between some pairs of land-use types, low absolute values of annual soil loss (2−3 orders of magnitude lower than the tolerable soil erosion rate [56]) suggest that the service of soil erosion control is delivered as long as soils are protected by vegetation cover, as exemplified in a review for the humid tropics [57]. However, it cannot be asserted that soil erosion is consistently negligible across the landscape and throughout time. We did not monitor soil loss on bare soil elements created by logging activities (e.g. dirt roads, landslides) or related to rubber tapping (e.g. walking tracks) that are known to contribute to erosion at the landscape scale disproportionately compared to their geographically restricted areas [58,59]. Nor did we monitor soil loss immediately after a major disturbance (e.g. burning or opening of logging tracks). Since extreme rainfall events that occur while soil is temporarily bare can lead to dramatic soil loss, soil loss monitoring before, during and after disturbance is required to follow soil erosion control during land-use change [60].

Our results on tree species diversity show that some areas of human-modified land-use types host high tree species richness (e.g. logged-over forests and old secondary regrowth forest). This has already been shown for various taxa and is a widely accepted phenomenon [6,8,54,61]. However, we also show that the tree species composition of natural forest is highly specific (cf. Fig 3), which strengthens the assertion that “primary forests are irreplaceable for sustaining tropical biodiversity” due to habitat restrictions of some highly sensitive or specialist species to such natural habitats [62,63].

Overall, in our study site, natural forests produce the highest levels of services across the landscape and this would lend support to land management strategies that promote their strict protection [64]. Schemes and financial mechanisms in this area that conserve one of these services (e.g. carbon with REDD+, a financial mechanism aiming at Reducing Emissions from Deforestation and Forest Degradation) would synergistically benefit the others, therefore increasing the effectiveness of natural forest protection. But correlations between services depend on the spatial resolution and the scope of a study [65–67]. Therefore, more primary data are needed for inferring that mechanisms targeting carbon conservation would necessarily maximize benefits for biodiversity and other ecosystem services at the regional scale.

### Logged-over forests and swidden agriculture system outperform monocultures in terms of service production

In addition to the need to preserve natural forests, another challenge is to maximize service production in human-modified land-use areas. With the noticeable exception of tree species richness and tree species composition, our data did not show significant differences in service production between logged-over forests, old rubber gardens and old secondary regrowth forests. Tree species richness is high in logged-over forests (one plot can simultaneously host a mixture of old growth and secondary species), but in such forests, aboveground carbon stocks are low. In contrast, aboveground carbon stocks can be large in some old rubber gardens and secondary regrowth forests, but tree species richness is low. Beyond the diversity of products from these land-use types, there is a complementarity in services produced by such human-modified landscapes. Despite continuous changes in land use and management, the diversification of human activities—farming, rubber tapping, and logging—ensures the sustained delivery of multiple services.

As anticipated, comparing our results with those in the literature emphasized that a mosaic of land uses produces far more services than do rubber or oil palm monocultures: biodiversity is greater [13], erosion is lower (especially when plantations are set up on steep slopes), and aboveground carbon stocks are more than twice as great [68].

Our results support the finding that logged-over forests in the study area are better than monocultures in terms of biodiversity conservation and ecosystem service production. Hopefully, the strong case made by this study and many others (e.g. [5,8]) will eventually raise awareness among decision-makers and land-use planners that logged-over forests are not just worth being converted but should be sustainably managed.

### Is the swidden system in Keluin close to a sustainability threshold?

We found that carbon storage and tree diversity increased along a successional recovery of the forest after initial clearance: levels of service production were lowest in food crop fields, intermediate in young rubber gardens and secondary regrowth areas, and highest in old rubber gardens and secondary regrowth forests. More striking was no trend for carbon storage in topsoil and the slow recovery of different ecosystem services, either in old rubber gardens or secondary regrowth forests. Mean time since last disturbance for our old secondary regrowth forest plots was 47 years, and yet species richness was still significantly lower than for natural forests. Similarly, we found low similarity in tree species composition between old secondary regrowth forest and natural forest plots.

From a meta-analysis of the recovery of plant biodiversity and carbon stocks in secondary forests, 50 years are enough for species richness to reach natural forest levels, but with only a very low proportion of native forest species (mean value: 26%), even in old stands [54]. The aboveground carbon stocks we found in old secondary regrowth forests (ca. half those of natural forests) are consistent with the literature but lay in the lower part of the range compiled by the meta-analysis that reports aboveground carbon stock of 50–70% pre-disturbance levels after ca. 50 years of forest recovery [54]. Old rubber gardens also showed lower values of aboveground carbon stocks than those found in the literature [69].

We found topsoil carbon stocks for food crop fields to be half those of natural forest. Our results are consistent with those of a meta-analysis that also showed that soil carbon stocks will eventually fully recover as croplands are allowed to revert to secondary forests [70]. Another study estimated that 40−50 years are needed for secondary forest soil carbon stocks to reach pre-disturbance levels [71]. Despite the mean time since last disturbance (42 years) being within this range, topsoil carbon in old secondary regrowth forests was far below the pre-disturbance levels in our study site.

One study that was also carried out in West Kalimantan found that an increasing number of cycles of cultivation and forest regrowth did not lead to total phosphorus decline, but had detrimental consequences for aboveground carbon sequestration [72,73]. The capacity of soils to recover carbon content after disturbances might also be reduced in plots where numerous rotations have already been done. The whole swidden agriculture system is sustainable if the condition that sufficient time is allowed for soil and vegetation to recover is met. Soil impoverishment related to reduction in rotation length is a serious threat likely to jeopardize the production of goods and services in the long-term from the traditional swidden system in the Keluin area.

## Conclusion

In such a rapidly transforming traditional rural landscape in northern Bornean, natural forests host highly unique tree species diversity, have the lowest erosion rate, and store significantly more carbon (in aboveground biomass and topsoil) than do any other land-use type. Logged-over forests provide services similar to natural forest, except for soil erosion control, which is jeopardized by the presence of the remaining decaying road network that leads to soil loss at the landscape level.

All land uses related to the swidden agriculture system largely outperform oil palm or rubber monocultures in terms of tree diversity, carbon storage, and soil erosion control. Natural and logged-over forests should be maintained or managed as an integral part of the swidden system, and landscape multifunctionality should be sustained as a safety net against the price volatility of traded goods (e.g. rubber, palm oil, timber, tengkawang oil), upon which the economy of monocrop systems is much more dependent.

Because of the congruence of services in natural forest, protection of their carbon stocks, for example through financial mechanisms such as REDD+, will synergistically benefit biodiversity and a wide range of other services provided to communities in this area. However, how such mechanisms could benefit communities must be carefully evaluated to counter the high opportunity cost of conversion to monocultures; these may generate greater income, but may also be more detrimental to the production of multiple ecosystem services.

Ecosystem service recovery time following initial slash-and-burn practices on the vegetation is longer in the study area than has been reported in the literature for similar study situations. As rotation length appears to be a key factor in the sustainability of swidden systems, it is critical to understand the socio-cultural and economic drivers of the reduction in rotation length and the potential feedback of this reduction on social–ecological systems. In the rapidly transforming socio-environmental context of this region, questions remain about the long-term persistence of the swidden agriculture system.

## Supporting Information

S1 Fig Individual-based rarefaction curves for every land-use type. Rarefaction level was the number of individuals surveyed in old rubber gardens (n = 449). Twelve plots (0.48 ha in total) were surveyed for fallows and rubber gardens (both young and old), and 25 plots (1 ha in total) for logged-over and natural forest.
(TIFF)

S1 Table Sampling design for vegetation and erosion plots. No vegetation plot was selected in food crop fields under the assumption that tree diversity and aboveground carbon would be null. The first plot size dimension is the dimension along the slope.
(XLSX)

S2 Table Vegetation plot features. Longitude and latitude of each plot are provided in decimal degrees (WSG84 datum). Plot mean diameter at breast height (DBH), height, wood specific gravity (WSG) and aboveground carbon (AGC) are also provided.
(XLSX)

S3 Table Erosion plot features. Longitude and latitude of each plot are provided in decimal degrees (WSG84 datum). Plot annual soil loss (ASL) and topsoil carbon (TSC) are also provided. ASL values in grey-tinted cells were discarded for analyses.
(XLSX)

S4 Table Statistical analyses on ecosystem service and tree diversity indicators. We used original indicator values when the distribution was normal and log_10_-transformed values otherwise. The vegetation set did not include food crop field plots under the assumption that tree diversity and aboveground carbon would be null. Depicted values are either test statistics (for Moran’s I, Lagrange Multiplier and the selected model) or model coefficients, and are presented along with information on statistical significance. For the Berger-Parker index, despite some spatial auto-correlation, the spatial dependence coefficient of the spatial error model was not significant. We therefore used a regular linear model and acknowledge that results for this indicator might be slightly biased due to spatial auto-correlation.
(XLSX)

S5 Table Species surveyed in each land-use type. Data from plots under the same land-use type (12 for young and old rubber gardens and fallows and 25 for logged-over and natural forests) were pooled together. IUCN conservation status is provided for each species (CR = Critically Endangered; EN = Endangered; VU = Vulnerable; LR/cd = Lower Risk: Conservation Dependent; LR/nt = Lower Risk: Near Threatened; DD = Data Deficient; LC or LR/lc = Least Concern). In order to be conservative, any species identified to the genus level only (e.g. *Aglaia* sp.3) or absent from the IUCN Red List was given a “LC” status.
(XLSX)
